# Addiction and the Brain-Disease Fallacy

**DOI:** 10.3389/fpsyt.2013.00141

**Published:** 2014-03-03

**Authors:** Sally Satel, Scott O. Lilienfeld

**Affiliations:** ^1^Yale University School of Medicine, New Haven, CT, USA; ^2^Department of Psychology, Emory University, Atlanta, GA, USA

**Keywords:** brain-disease fallacy, addiction, fMRI, Project HOPE, brain-disease model

## Abstract

**From Brainwashed: The Seductive Appeal of Mindless Neuroscience by Sally Satel and Scott Lilienfeld, copyright © 2013. Reprinted by permission of Basic Books, a member of The Perseus Books Group**.

The notion that addiction is a “brain disease” has become widespread and rarely challenged. The brain-disease model implies erroneously that the brain is necessarily the most important and useful level of analysis for understanding and treating addiction. This paper will explain the limits of over-medicalizing – while acknowledging a legitimate place for medication in the therapeutic repertoire – and why a broader perspective on the problems of the addicted person is essential to understanding addiction and to providing optimal care. In short, the brain-disease model obscures the dimension of choice in addiction, the capacity to respond to incentives, and also the essential fact people use drugs for reasons (as consistent with a self-medication hypothesis). The latter becomes obvious when patients become abstinent yet still struggle to assume rewarding lives in the realm of work and relationships. Thankfully, addicts can choose to recover and are not helpless victims of their own “hijacked brains.”

## Introduction

In 1970, high-grade heroin and opium flooded Southeast Asia. Military physicians in Vietnam estimated that nearly half of all U.S. Army enlisted men serving there had tried opium or heroin [(1), p. 1046], and between 10 and 25% of them were addicted. Deaths from overdoses soared. In May 1971, the crisis reached the front page of the *New York Times*: “G.I. Heroin Addiction Epidemic in Vietnam” (2). Fearful that the newly discharged veterans would join the ranks of junkies already bedeviling inner cities, President Richard Nixon commanded the military to begin drug testing. No one could board a plane home until he had passed a urine test. Those who failed could attend an army-sponsored detoxification program (3).

Operation Golden Flow, as the military called it, succeeded. As word of the new directive spread, most GIs stopped using narcotics. Almost all the soldiers who were detained passed the test on their second try (4). Once they were home, heroin lost its appeal. Opiates may have helped them endure a war’s alternating bouts of boredom and terror, but stateside, civilian life took precedence. The sordid drug culture, the high price of heroin, and fears of arrest discouraged use, veterans told Lee Robins, the Washington University sociologist who evaluated the testing program from 1972 to 1974 (5).

Robins’ findings were startling. Only 5% of the men who became addicted in Vietnam relapsed within 10 months after return, and just 12% relapsed briefly within 3 years. “This surprising rate of recovery even when re-exposed to narcotic drugs,” wrote Robins, “ran counter to the conventional wisdom that heroin is a drug which causes addicts to suffer intolerable craving that rapidly leads to re-addiction if re-exposed to the drug”(1). Scholars hailed the results as “revolutionary” and “path-breaking” [(6), p. 215]. The fact that addicts could quit heroin and remain drug-free overturned the belief that “once an addict, always an addict.”

Unfortunately, that lesson has faded into the past. By the mid-1990s, the truism “once an addict, always an addict” was back, repackaged with a new neurocentric twist: “Addiction is a chronic and relapsing brain disease” (7). It was promoted tirelessly by psychologist Alan I. Leshner, then the director of the National Institute on Drug Abuse (NIDA), the nation’s premier addiction research body and part of the National Institutes of Health, and is now the dominant view of addiction in the field (8). The brain-disease model is a staple of medical school education and drug counselor training and even appears in the antidrug lectures given to high-school students (9). Rehab patients learn that they have a chronic brain disease. And the American Society of Addiction Medicine, the largest professional group of physicians specializing in drug problems, calls addiction “a primary, chronic disease of brain reward, motivation, memory and related circuitry” (10). Drug czars under Presidents Bill Clinton, George W. Bush, and Barack Obama have all endorsed the brain-disease framework at one time or another (11). From being featured in a major documentary on HBO, on talk shows and *Law and Order*, and on the covers of *Time* and *Newsweek*, the brain-disease model has become dogma – and like all articles of faith, it is typically believed without question (12–15).

That may be good public relations, but it is bad public education. We also argue that it is fundamentally bad science. The brain-disease model of addiction is not a trivial rebranding of an age-old human problem. It plays to the assumption that if biological roots can be identified, then a person has a “disease.” And being afflicted means that the person cannot choose, control his or her life, or be held accountable. Now introduce brain imaging, which seems to serve up visual proof that addiction is a brain disease. But neurobiology is not destiny: the disruptions in neural mechanisms associated with addiction do constrain a person’s capacity for choice, but they do not destroy it. What’s more, training the spotlight too intently on the workings of the addicted brain leaves the addicted person in the shadows, distracting clinicians, policy makers, and sometimes patients themselves from other powerful psychological and environmental forces that exert strong influence on them.

## Disease, Mind, and Brain

For over three centuries in the United States, physicians, legal scholars, politicians, and the public have debated the nature of addiction: is it a defect of the will or of the body? A moral or a medical problem? (16) Such polarization should by now have exhausted itself. After all, mountains of evidence attest to the fact that addiction entails both biological alterations in the brain and in personal agency. But given what is at stake in these debates – namely, our deep cultural beliefs about self-control and about deficits in personal responsibility paired with concerns about what society owes to addicts and what it can expect of them – we must be very careful not to ascribe too much influence to the addict’s brain.

This is an opportune time to pause and clarify two potential sources of misunderstanding.

First, we do not address the question of whether addiction is a “disease.” With the potential exception of certain organic brain syndromes, the field of psychiatry recognizes “disorders” or syndromes, rather than diseases because the etiologies of mental illness are not yet well understood. So, addiction fits the notion of disorder insofar as persistent craving and/or continued, excessive use leads to dysfunctional behavior. We are more concerned with the very different issue of whether addiction is best construed as a *brain* disease or *brain* disorder. Addiction is typically associated with brain changes, to be sure, but in contrast to conventional brain pathologies, such as Alzheimer’s disease, those alterations rarely if ever preclude individuals’ capacity to alter their behavior based on foreseeable consequences. The term “brain disease,” which often implies a lack of control over behavior, obscures that crucial distinction. Moreover, although severe addictions are partly rooted in genetic predispositions that are themselves manifested in brain functioning, these conditions can be profitably understood at multiple levels of analysis (e.g., psychological, social, cultural) in addition to the neural level.

Second, our distinguishing between the addict’s brain and his or her mind does not imply an endorsement of substance dualism. That is, we do not believe that the mind and the brain are independent of each other or composed of different physical substances with consciousness existing in a spiritual world separate from the body. Few scientifically literate persons do. But, speaking of literacy, there is indeed value in examining the language people use when talking about the relationship between the brain and the mind. To say that the brain and the mind are “different” does not necessarily mean that the two are *materially* separate domains. Every subjective experience, from the ache of nostalgia to the frisson of a Christmas morning, corresponds to physical events in the brain. The mind – the realm of feelings, desires, ideas, memories, intentions, and subjective experience – is produced by the action of neurons and brain circuits. How else could it work?

Yet the mind is not identical with the matter that produces it; one cannot use the physical rules from the cellular level to completely predict activity at the psychological or behavioral level. Put somewhat differently, there is a fundamental difference between substance dualism and property dualism: the latter acknowledges that everything mental is ultimately produced by physical matter but allows for the fact that certain mental phenomena have different properties than neural phenomena (just as molecules themselves are not alive, but complex configurations of certain molecules can produce life).

At this time, one cannot rely on the brain alone to predict or understand everything important about human subjectivity or behavior. This is because many psychological phenomena are emergent properties of lower-order constituents such as neural circuits, neurons, proteins, and genes. “Constitutive” reductionism – reducing complex entities to the sum of their component parts to facilitate study – is not controversial in the scientific community; nor do we take issue with it. In his 2006 book, *An Argument for Mind*, Harvard University psychologist Jerome Kagan notes that the appreciation of an impressionistic painting requires far more than the sum of its parts (17). As the viewer slowly approaches Claude Monet’s painting of the Seine at Dawn, Kagan notes, there comes a moment when the scene dissolves into tiny patches of color. When we adopt the eliminative reductionist position, he writes, “the coherent psychological component vanishes” [(17), p. 213]. Some philosophers of mind take a different view. They speculate that such properties (the painting in full) will ultimately prove reducible to more basic elements (the paint) (18). They may prove correct. But for the foreseeable future, valuable information is often lost when descending from higher explanatory levels, such as mental states, to lower levels, such as neuronal systems.

The distinctions between substance and property dualism and between constitutive and greedy reductionism may appear arcane, but overlooking them can lead us to overestimate the explanatory power of neuroscientific findings. Take addiction, for example. The dominant view among researchers is that it is a “brain disease,” plain and simple. Without a doubt, chronic drug exposure often changes the brain, but knowledge of the neural mechanisms underlying addiction typically has less relevance to the treatment of drug addiction and alcoholism than the psychological and social causes. To be sure, intervening directly on the brain, say with medications such as methadone, can sometimes be of value too. But understanding the brain of addicts gives us only partial insight into why they become addicted and how they recover.

## Addiction as a “Brain Disease”

So, what exactly makes addiction a brain disease? “That addiction is tied to changes in brain structure and function is what makes it, fundamentally, a brain disease,” Leshner wrote in a now-landmark article in *Science* in 1997. But that can’t be right. Every experience changes the brain – from learning a new language to navigating a new city. It is certainly true that not all brain changes are equal; learning French is not the same as acquiring a crack habit. In addiction, intense activation of certain systems in the brain makes it difficult for users to quit. Genetic factors influence the intensity and quality of the subjective effect of the drug, as well as the potency of craving and the severity of withdrawal symptoms (19).

The process of addiction unfolds partly through the action of dopamine, one of the brain’s primary neurotransmitters. Normally, dopamine surges in the so-called reward pathway, or circuit, in the presence of food, sex, and other stimuli central to survival. Dopamine enhancement serves as a “learning signal” that prompts us to repeat eating, mating, and other pleasures. Over time, drugs come to mimic these natural stimuli. With every puff of a Marlboro, injection of heroin, or swig of Jim Beam, the learning signal in the reward pathway is strengthened, and in vulnerable users, these substances assume incentive properties reminiscent of food and sex.

“Salience” is the term that neuroscientists often use to describe the pull of substances on the addicted – it’s more of a sense of wanting, even needing, than liking. The development of salience has been traced to the nerve pathways that mediate the experience as they emerge from the underside of the brain, in an area called the ventral tegmentum, and sweep out to regions such as the nucleus accumbens, hippocampus, and prefrontal cortex, which are associated with reward, motivation, memory, judgment, inhibition, and planning.

Other nerve fibers travel from the prefrontal cortex, a region involved in judgment and inhibition, to parts of the brain that control behavior. As one psychiatrist put it memorably, “The war on drugs is a war between the hijacked reward pathways that push the person to want to use, and the frontal lobes, which try to keep the beast at bay” (20). Note the word “hijacked.” As shorthand for the usurpation of brain circuitry during the addiction process, it is a reasonable metaphor [(21), p. 1715].In the hands of brain-disease purists, though, “hijacking” has come to denote an all-or-nothing process, likened to a “switch in the brain” that, once flipped, affords no retreat for the addict (22). “It may start with the voluntary act of taking drugs,” Leshner said, “but once you’ve got (addiction), you can’t just tell the addict, ‘Stop,’ any more than you can tell the smoker ‘Don’t have emphysema”’ (23).

The reward circuit is also intimately involved in “cue-induced” craving. Such craving is a special species of desire that manifests itself in a sudden, intrusive urge to use brought on by “cues” associated with use. The mere clink of a whiskey bottle, a whiff of cigarette smoke, or a glimpse of an old drug buddy on the corner can set off an unbidden rush of yearning, fueled by dopamine surges. For the addict who is trying to quit, this is a tense feeling, not pleasurable at all. Because the rush of desire seems to come out of the blue, users may feel blindsided, helpless, and confused (24).

In a very impressive display of brain technology, scientists have used PET and fMRI scans to observe the neural correlates of craving. In a typical demonstration, addicts watch videos of people handling a crack pipe or needle, causing their prefrontal cortices, amygdala, and other structures to bloom with activity (25) (videos of neutral content, such as landscapes, induce no such response). Even in users who quit several months previously, neuronal alterations may persist, leaving them vulnerable to sudden, strong urges to use. The familiar “This is your brain on drugs” is still with us [this slogan was created in 1987 by an American drug-prevention charity. To illustrate how drugs affect the brain, an egg (representing the brain) was cracked on a sizzling frying pan (representing drugs). Result: fried brain]. Nowadays, however, the brain itself often substitutes for the fried egg.

## The Addiction Paradox

But that egg is not always sizzling. There is a surprising amount of lucid time in the daily life of addicts. In their classic 1969 study “Taking Care of Business: The Heroin User’s Life on the Street,” criminologists Preble and Casey (26) found that addicts spend only a small fraction of their days getting high. Most of their time is spent either working or hustling (27–29). The same is true for many cocaine addicts (30). We tend to think of them, at their worst, frantically gouging their skin with needles, jamming a new rock into a pipe every 15 min, or inhaling lines of powder. In the grip of such hunger, an addict cannot be expected blithely to get up and walk away.

These tumultuous states – with neuronal function severely disrupted – are the closest drug use comes to being beyond the user’s restraint. But in the days between binges, cocaine users worry about a host of everyday matters: should I find a different job? Enroll my kid in a better school? Kick that freeloading cousin off the couch for good? Attend a Narcotics Anonymous meeting, enter treatment, or register at a public clinic? It is during these stretches of relative calm that many addicts could make the decision to get help or quit on their own – and many of them do. But the decision to quit can be long in coming, far too long for those who destroy their health, families, or careers in the meantime.

The paradox at the heart of addiction is this: How can the capacity for choice coexist with self-destructiveness? “I’ve never come across a single person that was addicted that wanted to be addicted,” says neuroscientist Nora Volkow, who succeeded Leshner as director of NIDA in 2003 (31). Aristotle noted this paradox as well. He used the term Akrasia to denote an appetite or strong desire for pleasure that leads to actions that are harmful to our conscious wishes for our well-being (32). Hume spoke of “liberty” as being a “power of acting or not acting according to the determinations of the will” (33).

Indeed, how many of us have ever come across a heavy person who exercised his or her freedom expressly toward the goal of becoming fat? Many undesirable outcomes in life arrive incrementally. “We can imagine an addict choosing to get high each day, though not choosing to be an addict,” says psychologist Gene Heyman. “Yet choosing to get high each day makes one an addict” (34).

Let’s follow a typical trajectory to see how this dynamic plays out. In the early phase of addiction, drugs or alcohol become ever more appealing, while once-rewarding activities, such as relationships, work, or family, decline in value. The attraction of the drug starts to fade as consequences accrue – spending too much money, disappointing loved ones, attracting suspicion at work – but the drug still retains its allure because it blunts psychic pain, suppresses withdrawal symptoms, and douses intense craving [(35), p. 3]. Addicts find themselves torn between the reasons to use and reasons not to (36).

Sometimes a spasm of self-reproach or a flash of self-awareness tips the balance toward quitting. William S. Burroughs, an American novelist and heroin addict, calls this the “naked lunch” experience, “a frozen moment when everyone sees what is on the end of every fork” [(37), p. 199]. Lawford (38), himself in recovery from drugs and alcohol, edited a 2009 collection of essays called *Moments of Clarity* in which the actor Alec Baldwin, singer Judy Collins, and others recount the events that spurred their recoveries. Some quit on their own; others got professional help. A theme in each of their stories is a jolt to self-image: “This is not who I am, not who I want to be” (39). One recovered alcoholic describes the process: “You tear yourself apart, examine each individual piece, toss out the useless, rehabilitate the useful, and put your moral self back together again” (40). These are not the sentiments of people in helpless thrall to their diseased brains. Nor are these sentiments the luxury of memoirists. Patients have described similar experiences to us: “My God, I almost robbed someone!” “What kind of mother am I?” or “I swore I would never switch to the needle.”

## Long-Term Addiction is the Exception

And it turns out that quitting is the rule, not the exception – a fact worth acknowledging, given that the official NIDA formulation is that “addiction is a *chronic and relapsing* [italics added] brain disease.” The Epidemiological Catchment Area Study, done in the early 1980s, surveyed 19,000 people. Among those who had become dependent on drugs by age 24, more than half later reported not a single drug-related symptom. By age 37, roughly 75% reported no drug symptom. The National Comorbidity Survey, conducted between 1990 and 1992 and again between 2001 and 2003 and the National Epidemiologic Survey on Alcohol and Related Conditions, done between 2001 and 2002 with more than 43,000 subjects, found that 77 and 86% of people who said they had once been addicted to drugs or alcohol reported no substance problems during the year before the survey (41).

By comparison, people who were addicted within the year before the survey were more likely to have concurrent psychiatric disorders. Additionally, NIDA estimates that relapse rates of treated drug-addicted patients run from 40 to 60% (42). In other words, they are not representative of the universe of addicts. They are the hard cases – the chronic and relapsing patients. Yet these patients often make the biggest impressions on clinicians and shape their views of addiction, if only because clinicians are especially likely to encounter them.

Researchers and medical professionals err in generalizing from the sickest subset of people to the overall population of patients. This caveat applies across the medical spectrum. Just as the clinician wrongly assumes that all addicts must be like the recalcitrant ones who keep stumbling through the clinic doors, psychiatrists sometimes view people with schizophrenia as doomed to a life of dysfunction on the basis of their frequent encounters with those whose delusions and hallucinations don’t improve with treatment. The error of extrapolating liberally from these subsets of difficult patients is so common that statisticians Patricia and Jacob Cohen gave it a name: the “clinician’s illusion” (43).

## Intended Benefits of the Brain-Disease Model

Advocates of the brain-disease paradigm have good intentions. By placing addiction on an equal medical footing with more conventional brain disorders, such as Alzheimer’s and Parkinson’s, they want to create an image of addicts as victims of their own wayward neurochemistry. They hope that this portrayal will inspire insurance companies to expand coverage for addiction and politicians to allocate more funding for treatment [(44), p. 33]. And in the hands of Alan Leshner, the model has had real political utility (45). Before he was NIDA director, Leshner served as acting director of the National Institute of Mental Health. There, he saw how brain-disease “branding” could prompt Congress to act. “Mental health advocates started referring to schizophrenia as a ‘brain disease’ and showing brain scans to members of congress to get them to increase funding for research. It really worked,” he said (46).

Many experts credit the brain-disease narrative with enhancing the profile of their field. The late Bob Schuster, head of NIDA from 1986 to 1991, admitted that although he did not think of addiction as a disease, he was “happy for it to be conceptualized that way for pragmatic reasons… for selling it to Congress”(47). For decades, addiction research had been a low-status field, disparaged by other researchers as a soft science that studied drunks and junkies. Now the field of neuroscience was taking greater notice. “People recognize that certain decision makers and others are very impressed with molecular biology,” said Robert L. Balster, director of the Institute for Drug and Alcohol Studies at Virginia Commonwealth University (48).

Psychiatrist Jerome Jaffe, an eminent figure in the field and the first White House adviser on drugs (the precursor of the “drug czar”), sees the adoption of the brain-disease model as a tactical triumph and a scientific setback. “It was a useful way for particular agencies to convince Congress to raise the budgets (and) it has been very successful,” he said. Indeed, neuroimaging, neurobiological research, and medication development consume over half of the NIDA research budget. In light of the agency’s reach – it funds almost all substance-abuse research in the United States – it sets the national agenda regarding which research gets funded and therefore the nature of the data produced and the kinds of topics that investigators propose. But Jaffe argues that the brain-disease paradigm presents “a Faustian bargain – the price that one pays is that you don’t see all the other factors that interact (in addiction)” (3).

Many proponents of the brain-disease concept were deeply committed to dispelling the stigma surrounding addiction. Medicalizing the condition was a powerful way, they hoped, to rehabilitate addicts’ poor public image from the perception of undisciplined deadbeats to people struggling with an ailment. This approach had its roots in the world of mental health advocacy. Until the early 1980s, plenty of people blamed parents for their children’s serious mental problems. Then advocates began to publicize neuroscientific discoveries, demonstrating, for example, that schizophrenia is associated with abnormalities of brain structure and function. In this effort, brain imaging has served sufferers well, helping legitimize their symptoms by representing visually the illness in their brains (49–53). The idea, of course, was that these benefits would extend to addicts. But it turns out that it’s harder to destigmatize addiction.

## Shortcomings of the Neurocentric View of Addiction

For all its benign aspirations, there are numerous problems with the brain-disease model. On its face, it implies that the brain is the most important and useful level of analysis for understanding and treating addiction. Sometimes the model even equates addiction with a neurological illness, plain, and simple (10). Such neurocentrism has clinical consequences, downplaying the underlying psychological and social reasons that drive drug use.

Recovery is a project of the heart and mind. The person, not his or her autonomous brain, is the agent of recovery. Notably, Alcoholics Anonymous, the institution perhaps most responsible for popularizing the idea that addiction is a disease, employs the term as a metaphor for loss of control. Its founders in the 1930s were leery of using the word “disease” because they thought that it discounted the profound importance of personal growth and the cultivation of honesty and humility in achieving sobriety (54).

The brain-disease narrative misappropriates language better used to describe such conditions as multiple sclerosis or schizophrenia – afflictions of the brain that are neither brought on by the sufferer nor modifiable by the desire to be well. It offers false hope that an addict’s condition is completely amenable to a medical cure (much as pneumonia is to antibiotics). Finally, as we’ll see, it threatens to obscure the vast role of personal agency in perpetuating the cycle of use and relapse.

Addicts embarking on recovery often need to find new clean and sober friends, travel new routes home from work to avoid passing near their dealer’s street, or deposit their paycheck directly into a spouse’s account to keep from squandering money on drugs. A teacher trying to quit cocaine switched from using a chalkboard – the powdery chalk was too similar to cocaine – and had a whiteboard installed instead. An investment banker who loved injecting speedballs – a cocktail of cocaine and heroin in the same syringe – made himself wear long-sleeved shirts to prevent glimpses of his bare and inviting arms. Former smokers who want to quit need to make many fine adjustments, from not lingering at the table after meals to ridding their homes of the ever-present smell of smoke, removing car lighters, and so on.

Thomas Schelling, a 2005 Nobel laureate in economics, refers to these purposeful practices as self-binding (55). The great self-binder of myth was Odysseus. To keep himself from heeding the overpowering song of the sea sirens – the half-woman, half-bird creatures whose beautiful voices lured sailors to their deaths – Odysseus instructed his men to tie him to the mast of his ship (56). The famous Romantic English poet Samuel Taylor Coleridge, an opium addict, is said to have hired men to prevent him from entering a pharmacy to purchase opium (57). Today, one can hire a firm that will provide binding services. It imposes surprise urine tests on the client, collects evidence of attendance at AA meetings or treatment sessions, and sends a monthly status report (with the good or bad news) to another person, such as a parent, spouse, or boss (58).

Some addicts devise their own self-binding strategies. Others need the help of therapists, who teach them to identify and anticipate cues that trigger craving. Beyond the classic triad of people, places, and things, they come to realize that internal states, such as stress, bad moods, and boredom, can prompt drug urges (59).

Managing craving matters mightily in recovery, but it usually is not enough. Another very important truth is that an addict uses drugs or alcohol because they serve a purpose. Caroline Knapp, in her powerful 1996 memoir *Drinking: A Love Story*, recounted why she spent two decades of her life as an alcoholic: “You drank to drown out fear, to dilute anxiety and doubt and self-loathing and painful memories” (60). Knapp doesn’t describe an urge to drink so much as a need to drink. She was not manipulated by an alien desire but by something woven into her being. To say that Knapp’s problem was merely the effect of heavy drinking on her brain is to miss the true threat to her well-being: the brilliant but tormented Knapp herself.

Heroin and speed helped screenwriter Jerry Stahl, author of *Permanent Midnight*, attain “the soothing hiss of oblivion.” But when the drugs wore off, his vulnerabilities throbbed like a fresh surgical incision. In surveying his life, Stahl wrote, “Everything, bad or good, boil(ed) back to the decade on the needle, and the years before that imbibing everything from cocaine to Romilar, pot to percs, LSD to liquid meth and a pharmacy in between: a lifetime spent altering the single niggling fact that to be alive means being conscious” [(61), p. 3–6]. The negative states to which we refer are typically underlying problems with emotional distress, especially mood or anxiety. To be sure, repeated use of drugs such as alcohol and cocaine can exacerbate primary depressive and anxiety disorders in the long term (62), but in the short term, the user almost always feels relief. Given the common problem of “steep discounting” in addicts, it is not surprising that addicts will attend to the experiencing self at the expense of the future self.

Or take Lisa, a 37-year-old woman featured in an HBO documentary on addiction. When we meet her, Lisa is living in a rundown hotel room in Toronto and working as a prostitute. She sits on the bed and talks with the filmmaker behind the camera. Flipping her shiny brown hair and inspecting her well-kept nails, Lisa is animated as she boasts about how much she makes selling sex, how much she spends on cocaine, and the longed-for “oblivion” that drugs help her attain. When Lisa was filmed, she was healthy and engaging; she looked and talked like someone who had recently been abstinent but was back in the early stages of her next downward spiral. She had no interest in stopping things at this point. “Right now, I am in no position to go into recovery (this way of life) is working for me … I have money, drugs, business. I’m O.K.” To say that Lisa’s problem is the effect of cocaine on her brain is to miss the true threat to her well-being: Lisa herself. “I always use for a reason. It’s repressing what needs to be repressed,” she says (63). To be certain, not all drug use in the service of improving mood is dysfunctional. But Lisa, who had been in treatment several times, is representative of individuals whose drug use starts out as a controlled and effective attempt at self-soothing but eventually becomes all-consuming and interferes with her life.

These stories highlight one of the shortcomings of the neurocentric view of addiction. This perspective ignores the fact that many people are drawn to drugs because the substances temporarily quell their pain: persistent self-loathing, anxiety, alienation, deep-seated intolerance of stress or boredom, and pervasive loneliness. The brain-disease model is of little use here because it does not accommodate the emotional logic that triggers and sustains addiction (35, 64, 65).

## The Power of Incentives and Addicts as Choosing Beings

In December 1966, Leroy Powell of Austin, TX, USA, was convicted of public intoxication and fined $20 in a municipal court. Powell appealed the conviction to county court, where his lawyer argued that he suffered from “the disease of chronic alcoholism.” Powell’s public display of inebriation therefore was “not of his own volition,” and the fine constituted cruel and unusual punishment. A psychiatrist concurred, testifying that Powell was “powerless not to drink” (66).

Then Powell took the stand. On the morning of his trial, he had a drink at 8 a.m. that his lawyer gave to him, presumably to stave off morning tremors. Here is an excerpt from the cross-examination:

Q: You took that one [drink] at eight o’clock [a.m.] because you wanted to drink?

A: Yes, sir.

Q: And you knew that if you drank it, you could keep on drinking and get drunk?

A: Well, I was supposed to be here on trial, and I didn’t take but that one drink.

Q: You knew you had to be here this afternoon, but this morning you took one drink and then you knew that you couldn’t afford to drink anymore and come to court; is that right?

A: Yes, sir, that’s right.

Q: Because you knew what you would do if you kept drinking that you would finally pass out or be picked up?

A: Yes, sir.

Q: And you didn’t want that to happen to you today?

A: No, sir.

Q: Not today?

A: No, sir.

Q: So you only had one drink today?

A: Yes, sir (66).

The judge let stand Powell’s conviction for public intoxication. A second appeal followed, this time to the U.S. Supreme Court. It, too, affirmed the constitutionality of punishment for public intoxication. “We are unable to conclude,” said the court, “that chronic alcoholics in general, and Leroy Powell in particular, suffer from such an irresistible compulsion to drink and to get drunk in public that they are utterly unable to control their performance” (66).

For people like Powell who are not otherwise motivated to quit, consequences can play a powerful role in modifying behavior. Powell took only a single drink the morning of his trial because of foreseeable and meaningful consequences. Far from being unusual, his ability to curtail his drinking accords with a wealth of studies showing that people addicted to all kinds of drugs – nicotine, alcohol, cocaine, heroin, methamphetamines – can change in response to rewards or sanctions (67–69). Powell had surely experienced many alcohol-induced brain changes, but they did not keep him from making a choice that morning.

If Powell came before a judge today, his lawyer might well introduce a scan of his brain “craving” alcohol as evidence of his helplessness. If so, the judge would be wise to reject the scan as proof. After all, a judge, or anyone, can ponder scans of “addicted” brains all day, but he or she would never consider someone an addict unless that person behaves like one (70–73). As legal scholar Stephen Morse puts it, “actions speak louder than images” (74).

Consider the following fMRI experiment by researchers at Yale and Columbia. They found that the brains of smokers reporting a strong desire to smoke displayed enhanced activation of reward circuitry, as would be expected (75). But they also showed that subjects could reduce craving by considering the long-term consequences of smoking, such as cancer or emphysema, while observing videos depicting people smoking. When subjects did so, their brains displayed enhanced activity in areas of the prefrontal cortex associated with focusing, shifting attention, and controlling emotions. Simultaneously, activity in regions associated with reward, such as the ventral striatum, decreased (76).

Investigators at NIDA observed the same pattern when they asked cocaine users to inhibit their craving in response to cues. Subjects underwent PET scanning as they watched a video of people preparing drug paraphernalia and smoking crack cocaine. When researchers instructed the addicts to control their responses to the video, they observed inhibition of brain regions normally implicated in drug craving. When not deliberately suppressing their cravings, the addicts reported feeling their typical desire to use, and the PET scans revealed enhanced activation in brain regions that mediate craving (77).

These powerful findings illuminate the capacity for self-control in addicts. They also underscore the idea that addicts persist not because of an inability to control the desire to use but from a failure of motivation. Granted, summoning sustained motivation can be a great challenge: it takes a lot of energy and vigilance to resist craving, especially urges that ambush the addict unexpectedly. Studies on the regulation of craving also help distinguish behavior that people do not control from behavior that they cannot control. Imagine, by way of contrast, promising a reward to people with Alzheimer’s if they can keep their dementia from worsening. That would be both pointless and cruel because the kinds of brain changes intrinsic to dementia leave the sufferer resistant to rewards or penalties.

What Powell’s case showed was that even though he sustained brain changes, those changes did not prevent his behavior from being shaped by consequences. Contingency management – the technical term for the practice of adjusting consequences, including incentives – often succeeds with people who face serious losses, such as their livelihood, professional identity, or reputation. When addicted physicians come under the surveillance of their state medical boards and are subject to random urine testing, unannounced workplace visits, and frequent employer evaluations, they fare well: 70–90% are employed with their licenses intact 5 years later [(78), p. 165]. Likewise, scores of clinical trials show that addicts who know they will receive a reward, such as cash, gift certificates, or services, are nearly two to three times as likely to submit drug-free urine samples as addicts not offered rewards (79, 80).

Unfortunately, treatment programs are rarely in a position to offer cash or costly rewards. But the criminal justice system has an ample supply of incentives at its disposal and has been using such leverage for years. One of the most promising demonstrations of contingency management comes from Honolulu in the form of Project HOPE, Hawaii’s Opportunity Probation with Enforcement.

Project HOPE includes frequent random drug testing of offenders on probation. Those who test positive are subject to immediate and brief incarceration. Sanctions are fair and transparent: all offenders are treated equally, and everyone knows what will happen in case of an infraction. The judges express a heartfelt faith in offenders’ ability to succeed. These basic elements of HOPE’s contingency administration – swiftness, sureness, transparency, and fairness combined with expectation for achievement – are a potent prescription for behavior change in just about anyone.

Indeed, after 1 year of enrollment in Project HOPE, participants fared considerably better than probationers in a group who served as a comparison. They were 55% less likely to be arrested for a new crime and 53% less likely to have had their probation revoked. These results are even more impressive in light of the participants’ criminal histories and their heavy, chronic exposure to methamphetamine, which can impair aspects of cognitive function [on project HOPE, see (81); on the effects of methamphetamines, see (82–86)].

These findings join a vast body of experimental data attesting to the power of incentives to override the lure of drugs. Yet because the facts contradict the idea that addiction is analogous to Alzheimer’s disease, some HOPE personnel objected to incentives, arguing that addicts couldn’t be accountable for their behavior. Likewise, when researchers asked NIDA to consider reviewing HOPE in its formative years, the agency declined on the grounds that methamphetamine addicts are not capable of responding to incentives alone (87–91).

## Can Medicine “Cure” addiction?

The brain-disease model leads us down a narrow clinical path. Because it states that addiction is a “chronic and relapsing” condition, it diverts attention from promising behavioral therapies that challenge the inevitability of relapse by holding patients accountable for their choices. At the same time, because the model implies that addicts cannot stop using drugs until their brain chemistry returns to normal, it overemphasizes the value of brain-level solutions, such as pharmaceutical intervention. In 1997, Leshner ranked the search for a medication to treat methamphetamine addiction as a “top priority (23). A decade later, Volkow predicted, “We will be treating addiction as a disease (by 2018), and that means with medicine” (15).

The search for a magic bullet is folly – and even NIDA has given up hope of finding a wonder drug – but the brain-disease narrative continues to inspire unrealistic goals. When British pop star Amy Winehouse succumbed to her high-profile alcoholism in the summer of 2011, a *Psychology Today* columnist asked, “Could neuroscience have helped Amy Winehouse?”(92). The author answered in the affirmative, suggesting a dopamine-altering medication of the future because addiction “may be a brain problem that science can eventually solve.” Neuroscientist David Eagleman goes even further, asserting that “addiction can be reasonably viewed as a neurological problem that allows for medical solutions, just as pneumonia can be viewed as a lung problem” (93). But the analogy doesn’t hold up. Changing a behavior like addiction requires addicts to work hard to change their patterns of thought and behavior. In contrast, antibiotic cures for pneumonia work even if the patient is in a coma.

The hope of a medical treatment is the logical outgrowth of placing the brain at the center of the addictive process. Overall, success to date has been genuine but modest. When motivated patients take medications – especially patients already armed with relapse-prevention strategies and the support of family and friends – they can sometimes vault into sustained recovery. Methadone, a long-acting synthetic opiate taken once a day to prevent opiate withdrawal, has played a major role in treating addiction to heroin and painkillers since the 1960s (94). Still, to their counselors’ chagrin, up to half the patients in methadone clinics also fortify themselves with heroin, cocaine, or Valium-like tranquilizers called benzodiazepines, sold on the street (95). Despite three decades of effort, there is still no medication therapy for cocaine. Cocaine immunotherapy (popularly called a cocaine “vaccine”) to prevent cocaine molecules from entering the brain is now in development, but previews do not look promising for wide-scale use (96). Other types of medications include blocking agents, such as naltrexone for opiate addiction, which occupy neuronal receptors and blunt a drug’s effect (97). Aversive agents, such as Antabuse (disulfiram), cause people to feel nauseated and vomit when they ingest alcohol (98). They can be effective in some cases, although many individuals elect to stop taking them.

These medications are not the product of modern neuroscience; they were developed decades ago. Even a vaccine was sought in the 1970s, although today’s techniques are vastly more sophisticated. More recently, neuroscientists have collaborated with pharmacologists to develop medications to reverse or compensate for the pathological effects of drugs on the brain. The premise is that different components of addiction can be targeted by different medications. These components are the “reward” circuit (which mediates a strong desire to use and preoccupation with imminent use) and the craving mechanism associated with conditioned cues. Thus far, success has been elusive. Anticraving agents have shown some promise for alcoholics, but treatments for cocaine addiction have been disappointing (99–102).

Traditionally, pharmacologists have approached the treatment of alcoholics and addicts in the same way they address most psychiatric diseases: as a matter of reversing or compensating for neuropathology – in this case, the neural alteration resulting from repeated use. This is a logical approach, but instead of focusing almost exclusively on what is wrong in the brain, perhaps they should also investigate the ways in which addicts recover. Addicts find non-drug sources of interests and gratification that generate their own outpourings of dopamine; they practice self-binding and mindfulness exercises that make the prefrontal cortex better at controlling impulses. Relinquishing drugs and alcohol is accompanied by a shift in the brain’s valuation systems. How, and even whether, these dynamics will translate into pharmacotherapy is a complicated question, but perhaps the answer will spur discovery of more effective medications – not panaceas but helpful aids to hasten the process of recovery. Some proponents of the brain-disease model would say that emphasizing the role of choice in addiction is just another way to stigmatize addicts and justify penal responses over therapeutic ones. To this way of thinking, if we see the addict as a “chronic illness sufferer,” we will no longer view him or her as a “bad person” (23, 103). This sentiment echoes throughout the addiction community. “We can continue playing the blame game,” said Volkow in 2008 “Or we can parlay the transformative power of scientific discovery into a brighter future for addicted individuals” (104).

## False Choices: Sick or Bad

Sick brain versus flawed character? Biological determinism versus bad choices? Why must these be our only options? This black-and-white framing sets a rhetorical trap that shames us into siding with the brain-disease camp lest we appear cruel or uncaring. The bind, of course, is that it is impossible to understand addiction if one glosses over the reality that addicts do possess the capacity for choice and an understanding of consequences. Forcing a choice between “sick or bad” adds confusion, not clarity, to the long-standing debate over just how much to hold addicts responsible in ways that are beneficial to them and to the rest of society.

Although it makes no sense to incarcerate people for minor drug crimes, exempting addicts from social norms does not ensure them a brighter future. Stigmatization is a normal part of social interaction – a potent force in shaping behavior. Author Susan Cheever, a former alcoholic, coined a new word, “drunkenfreude,” to denote how the embarrassing antics of intoxicated friends and strangers keep her sober. “[Watching] other people get drunk helps me remember,” Cheever writes. “I learn from seeing what I don’t want and avoiding it” (105).

Too often, well-meaning family members and friends try to insulate individuals from the consequences of their behavior and thereby miss an important opportunity to help the addict quit. There is nothing unethical – and everything natural and socially adaptive – about condemning reckless and harmful acts. At the same time, because addicts are people who suffer, we must also provide effective care and support progressive approaches, such as Project HOPE. If we want to garner social and political support for addicts’ plight, the best way to do that is to develop the most effective modes of rehabilitation possible – not to advance a reductive and one-dimensional version of addiction.

And what of the efforts to destigmatize addiction through medicalization? Results are mixed. In some surveys of the public, well over half of respondents saw addiction as a “moral weakness” or “character flaw.” In others, over half to two-thirds classified it as a “disease.” An Indiana University study asked over 600 people whether they viewed alcoholism as the result of a genetic problem or chemical imbalance (i.e., a “neurobiological conception”) or as an outgrowth of “bad character” or “the way he or she was raised.” Those endorsing a neurobiological explanation rose from 38% in 1996 to 47% in 2006; the proportion endorsing psychiatric treatment increased from 61 to 79% (106–112).

Another study revealed an unexpected pattern over the past few decades. As people accepted a biological explanation for mental illness and substance-abuse, their desire for social distance from the mentally ill and addicted increased. Biological explanations also appear to foster pessimism about the likelihood of recovery and the effectiveness of treatment (113–121). This finding may seem counterintuitive. One might think that a biological explanation would be good news to a patient – and to be sure, some people with mental illness do indeed find it a relief. But when the patient’s affliction is addiction and there are no medical cures to restore an addict’s disrupted brain, emphasizing the biological dimension seems misguided.

The authors of the chronic-brain-disease narrative were inspired by discoveries about the effects of drugs on the brain. The promise of finding powerful antiaddiction medications seemed great. The maturing science of addiction biology would mean that once and for all, the condition would be taken seriously as an illness – a condition that began with the explicit, voluntary decision to try drugs but transitioned into an involuntary and uncontrollable state. This knowledge, they hoped, would sensitize policy makers and the public to the needs of addicts, including access to public treatment and better private insurance coverage. A softening of puritanical attitudes and an easing of punitive law enforcement were also on the agenda.

The mission was worthy, but the outcome has been less salutary. The neurocentric perspective encourages unwarranted optimism regarding pharmaceutical cures and oversells the need for professional help. It labels as “chronic” a condition that typically remits in early adulthood. The brain-disease story gives short shrift to the reality that substances serve a purpose in addicts’ lives and that neurobiological changes induced by alcohol and drugs can be overridden.

Like many misleading metaphors, the brain-disease model contains some truth. There is a genetic influence on alcoholism and other addictions, and prolonged substance-abuse often damages brain structures that mediate self-governance. Yet the problem with the brain-disease model is its misplaced emphasis on biology as the star feature of addiction and its relegation of psychological and behavioral elements to at best supporting roles. “If the brain is the core of the problem, attending to the brain needs to be a core part of the solution,” as Leshner once put it (7). The clinical reality is just the opposite: The most effective interventions aim not at the brain but at the person. It’s the *minds* of addicts that contain the stories of how addiction happens, why people continue to use drugs, and, if they decide to stop, how they manage to do so. This deeply personal history can’t be understood exclusively by inspecting neural circuitry.

## Beyond the Brain

In the end, the most useful definition of addiction is a descriptive one, such as this: Addiction is a behavior marked by repeated use despite destructive consequences and by difficulty quitting not withstanding the user’s resolution to do so. This “definition” isn’t theoretical; it explains nothing about why one “gets” addiction – and how could it offer a satisfying causal account when there are multiple levels at which the process can be understood? Our proposed definition merely states an observable fact about the behavior generally recognized as addiction. That’s a good thing because a blank explanatory slate (unbiased by biological orientation or any other theoretical model) inspires broad-minded thinking about research, treatment, and policy. Is there room for neuroscience in this tableau? Of course. Brain research is yielding valuable information about the neural mechanisms associated with desire, compulsion, and self-control – discoveries that may one day be better harnessed for clinical use. But the daily work of recovery, whether or not it is abetted by medication, is a human process that is most effectively pursued in the idiom of purposeful action, meaning, choice, and consequence.

## Conflict of Interest Statement

The authors declare that the research was conducted in the absence of any commercial or financial relationships that could be construed as a potential conflict of interest.
